# The Relationship between Different Assays for Detection and Quantification of Amyloid Beta 42 in Human Cerebrospinal Fluid

**DOI:** 10.1155/2012/984746

**Published:** 2012-05-08

**Authors:** Teresa A. Ellis, Jinhe Li, David LeBlond, Jeffrey F. Waring

**Affiliations:** ^1^Neuroscience Biomarkers Group, Abbott Laboratories, Global Pharmaceutical Research and Abbot Park, Development, IL 60064-6123, USA; ^2^Exploratory Statistics, Abbott Laboratories, Global Pharmaceutical Research and Abbot Park, Development, IL 60064-6123, USA

## Abstract

Alzheimer's disease (AD), which is characterized by a degeneration of neurons and their synapses, is one of the most common forms of dementia. CSF levels of amyloid *β*_42_ (A*β*_42_) have been recognized as a strong candidate to serve as an AD biomarker. There are a number of commercial assays that are routinely employed for measuring A*β*_42_; however, these assays give diverse ranges for the absolute levels of CSF A*β*_42_. In order to employ CSF A*β*_42_ as a biomarker across multiple laboratories, studies need to be performed to understand the relationship between the different platforms. We have analyzed CSF samples from both diseased and nondiseased subjects with two different widely used assay platforms. The results showed that different values for the levels of CSF A*β*_42_ were reported, depending on the assay used. Nonetheless, both assays clearly demonstrated statistically significant differences in the levels of A*β*_42_ in CSF from AD relative to age-matched controls (AMC). This paper provides essential data for establishing the relationship between these assays and provides an important step towards the validation of A*β*_42_ as a biomarker for AD.

## 1. Introduction

Alzheimer's disease (AD) is the most common neurodegenerative disorder. Because the disease is often difficult to detect and diagnose at an early stage, a tremendous need exists for the identification and characterization of biomarkers that can be used to diagnose early-stage AD, or for monitoring new therapies for AD in clinical trials. Much interest has been generated regarding the use of CSF A*β*_42_ as a biomarker for diagnosing and tracking AD progression [1, 2]. Several different commercially available assays for measuring A*β*_42_ are currently employed across laboratories. These assays give diverse values for the levels of CSF A*β*_42_ [3, 4]. The relationships between the reported CSF A*β*_42_ values from these different assays are unclear, but researchers agree in the importance of standardizing assays for CSF A*β*_42_ [4].

Schoonenboom et al. compared the A*β*_42_ CSF measurements from two widely used A*β*_42_ ELISA assays using the same CSF sample. Our data extends their findings by directly comparing human CSF samples from both diseased and non diseased subjects with two different widely-used assay platforms, one of which uses colorimetric detection, while the other employs electrochemiluminescence (ECL) detection. Comparisons were made to determine if the assays gave similar values and were able to distinguish CSF from Alzheimer's subjects or age-matched controls based.

## 2. Materials and Methods

### 2.1. Subjects

Human cerebrospinal fluid (CSF) samples were purchased from Precision Med, Inc. (San Diego, CA), which is in compliance with all applicable rules and regulations for human sample collection and dissemination. CSF samples from 23 individuals with AD (14 males, 9 females, average age 78, average MMSE score 17) and 22 age-matched controls (11 males, 11 females, average age 77) were tested. To avoid any effects from multiple freeze-thaw cycles, 60 *μ*L aliquots of each CSF sample were placed in randomly assigned wells in a lo-bind polypropylene tray (Micronic North America, McMurray, PA). CSF was stored at −80°C until analysis. All samples were assayed in duplicate in both A*β*_42_ assays, conducted by the same experienced scientist.

### 2.2. CSF Analysis

Innotest *β*-Amyloid_1–42_ (Innogenetics, Alpharetta, GA) is a solid-phase ELISA for measuring the levels in human CSF utilizing colorimetric detection with peroxidase-labeled streptavidin. The ELISA was performed as outlined by the manufacturer's instructions. Absorbance at 450 nm was measured on the SpectraMax M2 (Molecular Devices, Sunnyvale, CA), and analysis was preformed using Softmax 5.2 software. The limit of detection (LOD) of 50 pg/mL was calculated by the manufacture as the mean of 8 determinations of the sample diluent.

MSD 96-well MULTI-ARRAY Human (6E10) A*β*_*x*-42_ assay (MesoScale Discovery, Gaithersburg, MD) was preformed as outlined by the manufacturer's instructions. Analysis was performed using MSD workbench version 3.0.17.3 (MSD, Gaithersburg, MD). An LOD of approximately 20 pg/mL was calculated by the manufacture based on data obtained from 4 different product lots (Table 1). All calibrators were prepared in siliconized polypropylene tubes (Sigma-Aldrich, St. Louis, MO), and CSF samples were diluted in 96-well u-bottom polypropylene plates (Costar, Lowell, MA).

### 2.3. Statistical Analysis

All statistical analyses and graphics were performed using either SAS JMP version 8 or R version 2.9.0. A *P* value < 0.05 was taken to indicate statistical significance. Statistical testing for differences in mean A*β*_42_ level among the two diagnosis groups (AD and AMC) was made using a two-sided, two-sample *t*-test.

## 3. Results

**Figure 1:**
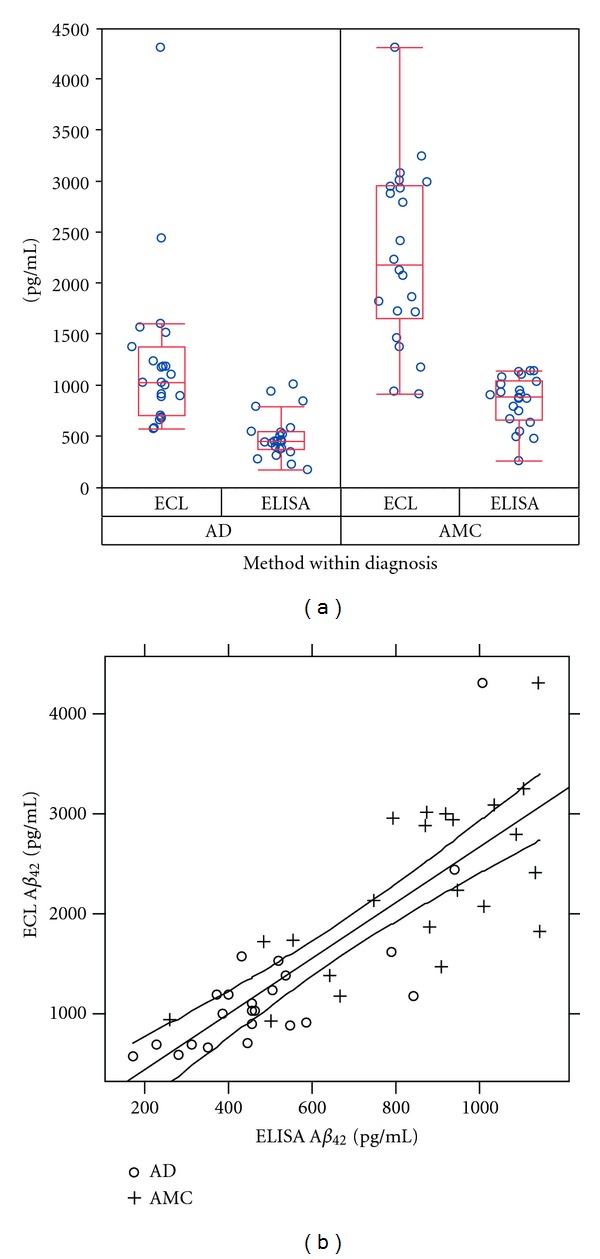
(a) CSF A*β*_42_ levels measured in both ELISA and ECL assay. Box plots of CSF A*β*_42_ levels from ELISA and ECL separated by diagnosis (AD and AMC) and method of detection. Boxes represent the interquartile ranges: bottom of box, black dot, and top of box indicate the 25, 50, and 75th percentile of the data. The whiskers (dashed bars) extend to the most extreme data points, which are no more than 1.5 times the height of the box away from the top or bottom of the box. (b) Correlation between ELISA and ECL. Correlation coefficients *r* = 0.819.

The A*β*_1–42_ ELISA standard curve showed a dynamic range of 125 to 2000 pg/mL, with an LOD of 50 pg/mL, and the average CV based on sample duplicates was 3.9%. The A*β*_*x*-42_ ECL assay standard curve showed a dynamic range of 12 to 3000 pg/mL, with an LOD of approximately 20 pg/mL, and the average CV based on sample duplicates was 7.1% (data not shown). The A*β*_42_ levels in the 45 CSF samples determined by ELISA and ECL assay are shown in Figure 1(a). The A*β*_42_ levels of all the CSF samples were above LODs in both assays. The means of A*β*_1–42_ measured by ELISA were significantly lower in AD patients compared with AMC (500.4 versus 848.3 pg/mL, resp.), (*P* < 0.0001*). The average CV based on sample duplicates was 6%. Mean A*β*_*x*-42_ measured by ECL assay were also significantly lower in AD patients compared with AMC (1235.8 versus 2280.5 pg/mL, resp.) (*P* < 0.0001*). The average CV based on sample duplicates was 8.4%. Although absolute concentrations varied between the ELISA and ECL, the correlation coefficient for CSF A*β*_42_ was *r* = 0.819 and highly significant (*P* < 0.0001) (Figure 1(b)).

## 4. Discussion

We provide here a direct comparison between two commonly used assays, ELISA and ECL assay, in measuring A*β*_42_ levels in human CSF. Both the ELISA and ECL assay showed that A*β*_42_ was higher in the AMC than the AD group. These observations in subject differentiation are similar to other published reports [1, 4–6].

We observed that in general A*β*_42_ levels were 2.6-fold higher in the ECL assay relative to the ELISA. The observed differences may be related to a number of factors including the matrix (i.e., different assay dilution buffers and reagents), the purity of the calibrators, and differences in the affinity of the capture and detection antibodies [3, 7]. The ELISA assay uses monoclonal antibody 21F12 as the capture antibody, which recognizes A*β*_1–42_. In contrast, ECL assay uses an undisclosed antibody, which recognizes A*β*_*x*-42_ as the capture antibody. There is also a lack of synchronization between the two assays due to the difference between the two calibrators. We conducted an experiment swapping the calibrators between the two kits. Both assays detected the other kits' calibrator; however, the % recovery was not at an acceptable level (data not shown).

Although both are plate-based methods, the detection technologies for measuring the CSF A*β*_42_ concentrations are different and could be a contributing factor to the underlying difference. The ELISA signal is detected with a peroxidase-labeled streptavidin antibody and the result is colorimetric, which provides the desired sensitivity but less dynamic range. The ECL signal is detected by incorporating a SULFO-TAG labeled antibody that emits light upon electrochemical stimulation initiated at the electrode, which provides sensitivity similar to the ELISA but a broader dynamic range.

## 5. Conclusion

Currently, quality control efforts are under investigation to evaluate interlaboratory variance components and to aid in the standardization of CSF A*β*_42_ measurements [1]. In addition, larger harmonization studies are needed that include the assays studied here as well as other manufactures assays for the measurement of A*β*_42_ in human CSF. The present study provides an important first step by comparing and establishing the relationship between two widely used platforms for measuring A*β*_42_.

## Figures and Tables

**Table 1:** 

	ELISA	EC	Ratio
	pg/mL	pg/mL	ECL/ELISA
AD (*n* = 23) Mean ± SE	500.4 ± 44.6	1235.8 ± 165.7	2.5
AMC (*n* = 22) Mean ± SE	848.3 ± 51.9	2280.5 ± 184.6	2.7
LOD	50 pg/mL	10–20 pg/mL	

Average			2.6
